# Tau overexpression impairs neuronal endocytosis by decreasing the GTPase dynamin 1 through the miR‐132/MeCP2 pathway

**DOI:** 10.1111/acel.12929

**Published:** 2019-02-27

**Authors:** Ao‐Ji Xie, Tong‐Yao Hou, Wan Xiong, He‐Zhou Huang, Jie Zheng, Ke Li, Heng‐Ye Man, Ya‐Zhuo Hu, Zhi‐Tao Han, Hong‐Hong Zhang, Na Wei, Jian‐Zhi Wang, Dan Liu, Youming Lu, Ling‐Qiang Zhu

**Affiliations:** ^1^ Department of Pathophysiology, School of Basic Medicine, Tongji Medical College Huazhong University of Science and Technology Wuhan China; ^2^ Department of Biology Boston University Boston Massachusetts; ^3^ Beijing Key Laboratory of Aging and Geriatrics, National Clinical Research Center for Geriatric Disease, Institute of Geriatrics Chinese PLA General Hospital & Chinese PLA Medical Academy Beijing China; ^4^ Department of Pathology The First Affiliated Hospital of Zhengzhou University Zhengzhou China; ^5^ The Institute for Brain Research, Collaborative Innovation Center for Brain Science Huazhong University of Science and Technology Wuhan China

**Keywords:** dynamin 1, endocytosis, miR‐132, Tau

## Abstract

Tauopathies are a class of neurodegenerative diseases that are characterized by pathological aggregation of tau protein, which is accompanied by synaptic disorders. However, the role of tau in endocytosis, a fundamental process in synaptic transmission, remains elusive. Here, we report that forced expression of human tau (hTau) in mouse cortical neurons impairs endocytosis by decreasing the level of the GTPase dynamin 1 *via* disruption of the miR‐132‐MeCP2 pathway; this process can also be detected in the brains of Alzheimer's patients and hTau mice. Our results provide evidence for a novel role of tau in the regulation of presynaptic function.

Pathologically aggregated tau is recognized as the dominant component of neurofibrillary tangles (NFTs), which constitute the characteristic pathological hallmark of tauopathies, including Alzheimer's disease (AD). Under normal conditions, tau is predominantly distributed in the axon, the main presynaptic loci. Soluble pathological tau in the entorhinal cortex leads to presynaptic deficits in a model of early AD (Polydoro et al., 2014). Tau binds to synaptic vesicles and interferes with their mobility and release rate (Zhou et al., 2017). These data suggest a critical role for tau in presynaptic function. Clathrin‐mediated endocytosis has been implicated in the recycling of synaptic vesicles, which is attenuated in the brains of AD patients and in Aβ‐challenged neurons in culture (Kelly & Ferreira, 2007). However, whether tau affects synaptic endocytosis remains unknown.

To understand the potential role of tau in neuronal endocytosis, we forced the expression of full‐length human tau in cultured neurons and examined the internalization of transferrin (Tf)‐546 (Figure 1a). We found that hTau‐overexpressing neurons displayed a severe deficiency in Tf uptake (Figure 1a,b) and an increase in membrane vesicular glutamate transporter 1 (VGlut1) and synaptophysin (Supporting Information Figure S1). However, hTau overexpression decreased only the mRNA and protein levels of dynamin 1 (Figure 1c–e). No significant differences were found in the mRNA or protein level of clathrin. Moreover, the tau‐induced decrease in dynamin 1 is dose dependent (Figure 1f,g). In the brains of human tau‐overexpressing mice (3 months, Figure 1h–j; 12 months, Supporting Information Figure S2) and AD patients (Figure 1k,l), decreased expression of dynamin 1 was also detected, suggesting that tau overexpression impairs synaptic endocytosis by suppressing dynamin 1 transcription.

**Figure 1:**
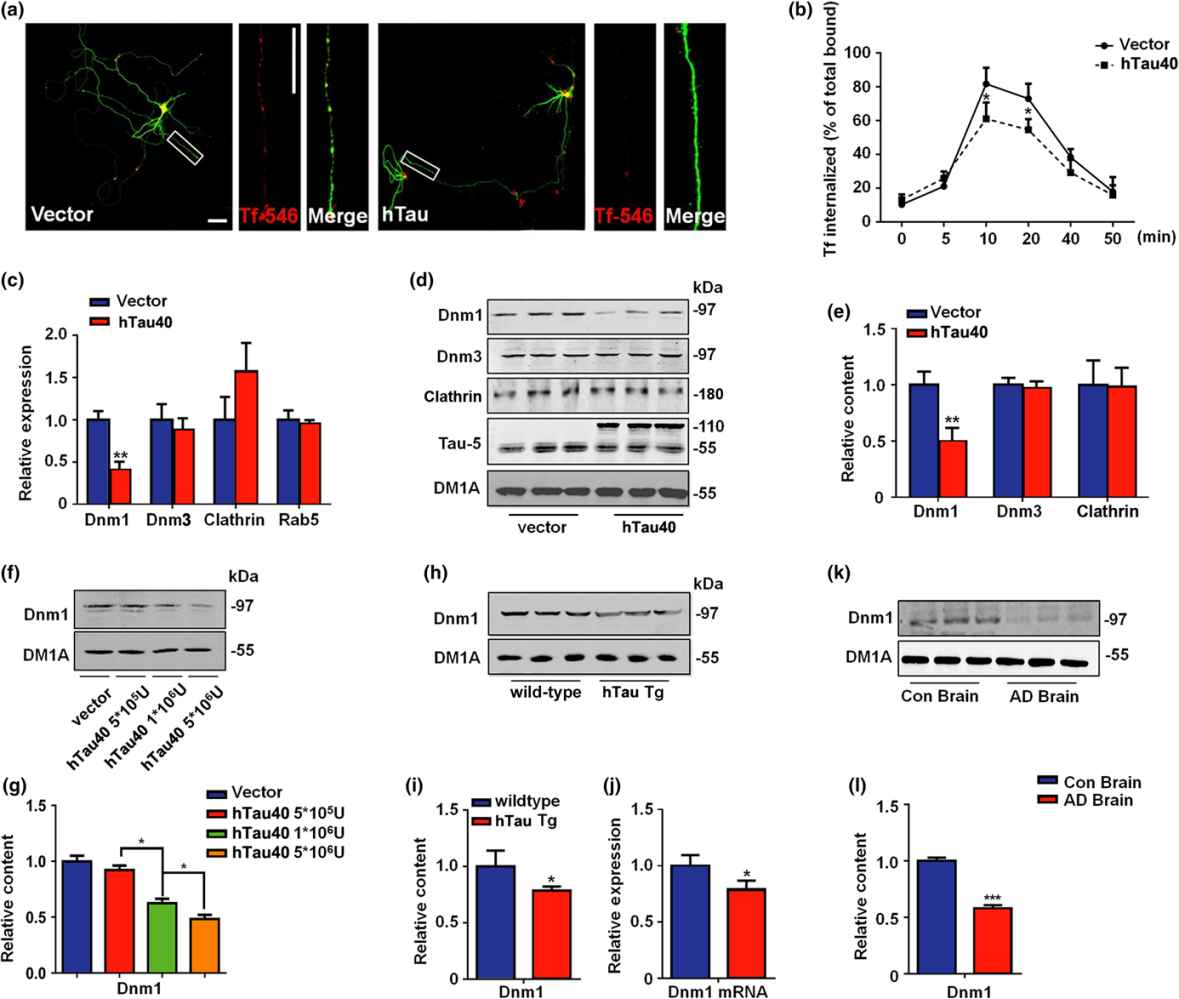
Tau interrupts synaptic endocytosis by decreasing dynamin 1. (a) Primary cortical neurons were infected with lentivirus packed hTau‐EGFP or EGFP at DIV7, and the Transferrin (Tf‐546) uptake experiments were performed at 72 hr later. The red color indicates the internalized Transferrin. Bar = 50 μm. *N* = 5. (b) The effects of hTau on Tf‐546 endocytosis were detected in several time points. *N* = 5. (c) The neurons were treated as above, and the mRNA of dynamin 1 (Dnm1), dynamin 3 (Dnm3), clathrin, and Rab5 were detected. *N* = 5. **p < 0.01, vs.* vector.* (d) The representative blots of dynamin1, dynamin3, clathrin, and Tau5 and (e) the quantification. *N* = 5. **p < 0.01, vs.* vector.* (f) The representative blots of dynamin1 in neurons that treated with different hTau lentivirus dilutions and (g) the quantification. *N* = 5.*p < 0.05*, *vs.* vector.* (h) The representative blots of dynamin1 in the cortex of 12 weeks hTau transgenic mice and their wild‐type and (i) the quantification. *N* = 6. *p < 0.05, vs.* wild‐type.* (j) The dynamin1 mRNA level in the cortex of 12 weeks hTau transgenic and age‐matched wild‐type mice. *N* = 5. *p < 0.05, vs.* wild‐type.* (k) The representative blots of dynamin1 in AD brain and control brain, and (l) the quantification. *N* = 5. *p < 0.05*, *vs.* control*

Methyl‐CpG‐binding protein 2 (MeCP2) was reported to bind to the promoter regions of dynamin 1 (Gibson et al., 2010), and MeCP2 overexpression reduced both the mRNA and protein levels of dynamin 1 in cultured neurons (Supporting Information Figure S3), implying that dynamin 1 is transcriptionally regulated by MeCP2. Furthermore, we found that the MeCP2 protein levels were increased in hTau‐overexpressing neurons (Figure 2a,b), in the cortex of hTau transgenic mice (Figure 2d,e) and in the brains of AD patients (Figure 2g,h) but that the mRNA levels were unchanged (Figure 2c,f), which indicates that the upregulation of MeCP2 induced by tau overexpression occurs posttranscriptionally. Only miR‐132 is downregulated in hTau‐overexpressing neurons and hTau mice (Figure 2i,j). A luciferase reporter experiment confirmed the direct regulation of MeCP2 by miR‐132 (Supporting Information Figure S4). Importantly, administration of miR‐132 mimics not only rescued dynamin 1 loss and MeCP2 upregulation both in vitro (Figure 2k–m) and in vivo (Supporting Information Figure S5) but also restored the synaptic endocytosis deficits induced by hTau overexpression (Figure 2n,o). Together, these results indicate that the miR‐132/MeCP2/dynamin 1 pathway participates in hTau‐induced endocytosis deficiency.

**Figure 2:**
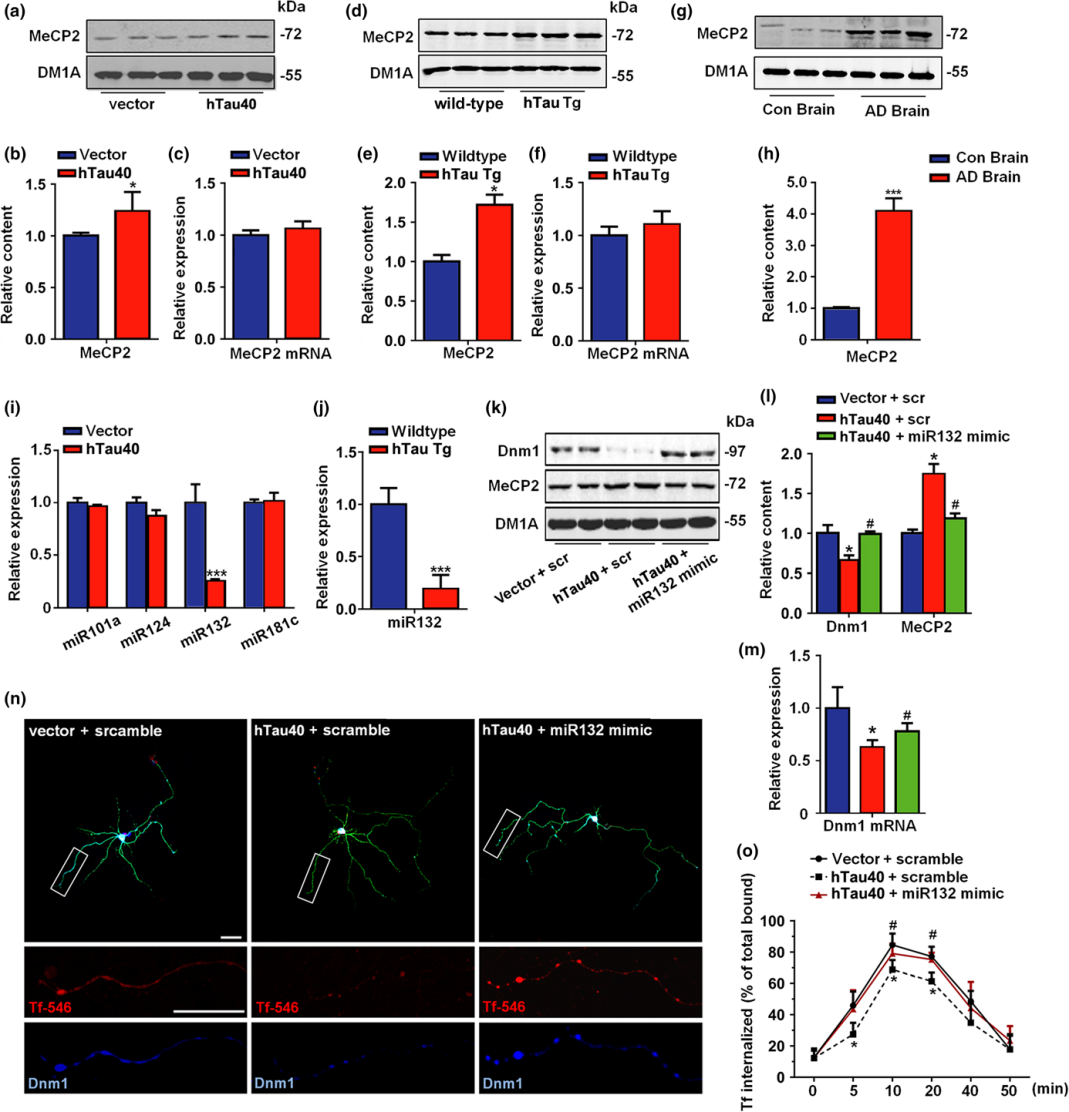
MiR‐132/MeCP2 signal is involved in tau‐induced synaptic endocytosis deficits. (a) Neurons were infected with hTau virus or control, the representative blot of MeCP2 was shown and (b) the quantification. (c) mRNA level of MeCP2 in neurons treated above. *N* = 5. *p < 0.05, vs.* vector.* (d) The representative blots of MeCP2 in 12 weeks hTau transgenic mice cortex and their wild‐type and (e) the quantification. (f) The mRNA level of MeCP2 from above samples. *N* = 6. *p < 0.05*, *vs.* wild‐type.* (g) The representative blots of MeCP2 in AD brain samples and (h) the quantification. *N* = 5. ***p < 0.001*, *vs.* con.* (i) The levels of different microRNAs in hTau neurons. *N* = 5. ***p < 0.001, vs.* vector.* (j) The level of miR132 in the cortex of 12 weeks hTau transgenic mice and the wild‐type. *N* = 5. ***p < 0.001*, *vs.* wild‐type.* (k) The representative blots of dynamin1, MeCP2 in primary cortical neurons transfected with vector, hTau or hTau +miR132 mimics and (l) the quantification. *N* = 5. *p < 0.05*, *vs.* vector. ^#^p < 0.05, *vs.* hTau neurons.* (m) The mRNA level of dynamin1 in the neurons treated as described in k. *p < 0.05*, *vs.* vector. ^#^p < *0.05, vs.* hTau neurons.* (n) Representative images or (o) timeline curve for the effects of miR‐132 on Tf‐546 endocytosis in hTau neurons. Bars = 50 μm. *N* = 5. *p < 0.05, vs.* vector. ^#^p < 0.05, *vs.* hTau neurons*

Tauopathy, especially the abnormal hyperphosphorylation and aggregation of tau, is one of the most prominent pathological hallmarks of AD. hTau mice (those that overexpress human tau) develop hyperphosphorylated, conformationally altered tau aggregates in the cell bodies and dendrites of neurons, a phenotype that is recognized to closely recapitulate a type of tau pathology found in early AD (Andorfer et al., 2003). As is the case with several other miRNAs that are deregulated in AD, the expression of miR‐132 is negatively correlated with tau pathology (Smith et al., 2015). Loss of miR‐132 has been found to occur in many tau‐related diseases (Salta & De Strooper, 2017), indicating its potential role in mediating tau‐related neurodegeneration. Here, we found that tau overexpression led to the loss of miR‐132, while miR‐132 deficiency in mice led to increased tau expression, phosphorylation, and aggregation. Moreover, in the hTau mouse brain, the MeCP2 level is increased, and MeCP2 can regulate tau expression and phosphorylation and thus contribute to tauopathy in AD (Maphis et al., 2017). These studies strongly suggest a vicious cycle of miR‐132‐tau or tau‐miR‐132‐MeCP2‐tau abnormalities in the tauopathies.

Taken together, the results of our study demonstrate that tau interferes with neuronal endocytosis through the miR‐132‐MeCP2‐dynamin 1 pathway, and they provide a possible mechanism of tau‐induced neuronal dysfunction and neurodegenerative pathogenesis.

## CONFLICT OF INTEREST

None declared.
