# Supplementary material for: Tau overexpression impairs neuronal endocytosis by decreasing the GTPase dynamin 1 through the miR‐132/MeCP2 pathway
Source: Aging Cell. 2019 Feb 27;18(3):e12929. doi: 10.1111/acel.12929 (PMC6516177; doi:10.1111/acel.12929)
Supplement: Supplementary file 1 [file ACEL-18-e12929-s001.doc]

**Supporting information**


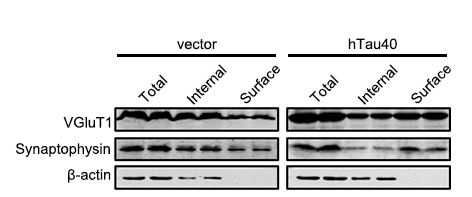


**Supplementary Figure 1. hTau-overexpressed neurons displayed an increase in the plasma membrane levels of VGluT 1 and synaptophysin .**

Cell surface biotinylation experiments showed that the fractions of VGluT 1 and synaptophysin that were surface exposed (biotinylated) were greatly increased in hTau-overexpressed neurons. As expected, actin was exclusively detected in the internal (nonbiotinylated) fraction.


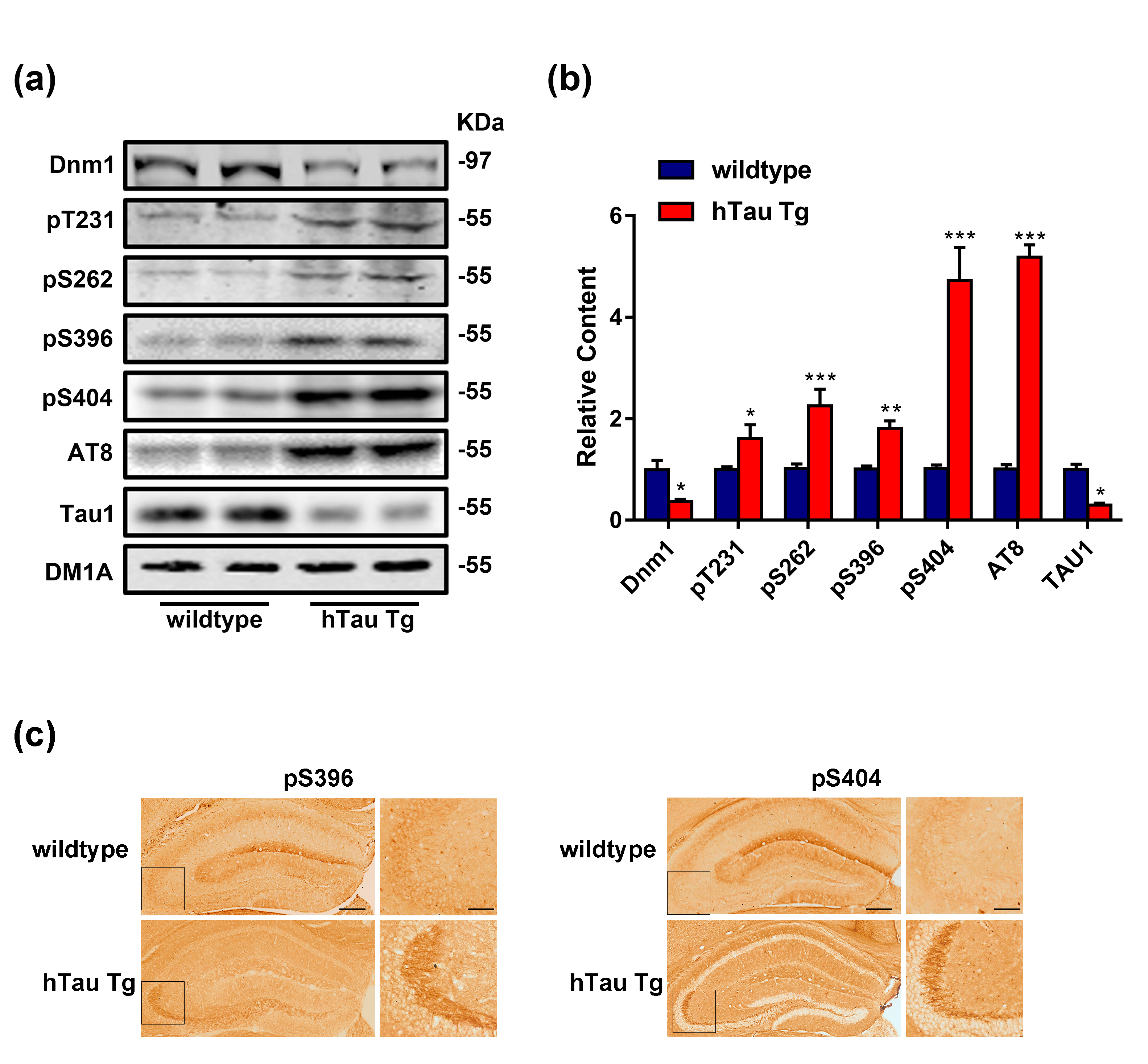


**Supplementary Figure 2. Decreased expression of dynamin 1 was detected in the brains of human tau-overexpressed mice (12 month, tauopathy).**

(a) The representative blots of Dynamin1, pT231, pS262, pS396, pS404, AT8, and Tau1 in the cortex of 12 months wildtype mice or human tau-overexpressed mice. (b) The quantitative analysis. N=6. **p<0.05, **p<0.01,***p<0.001, vs wildtype.* (c) Brain sections including the hippocampal formation were immunostained with pS396 and pS404. The CA3 regions are magnified in the right panel. Scale bar=300 μm for left panel and scale bar=60 μm for right panel.


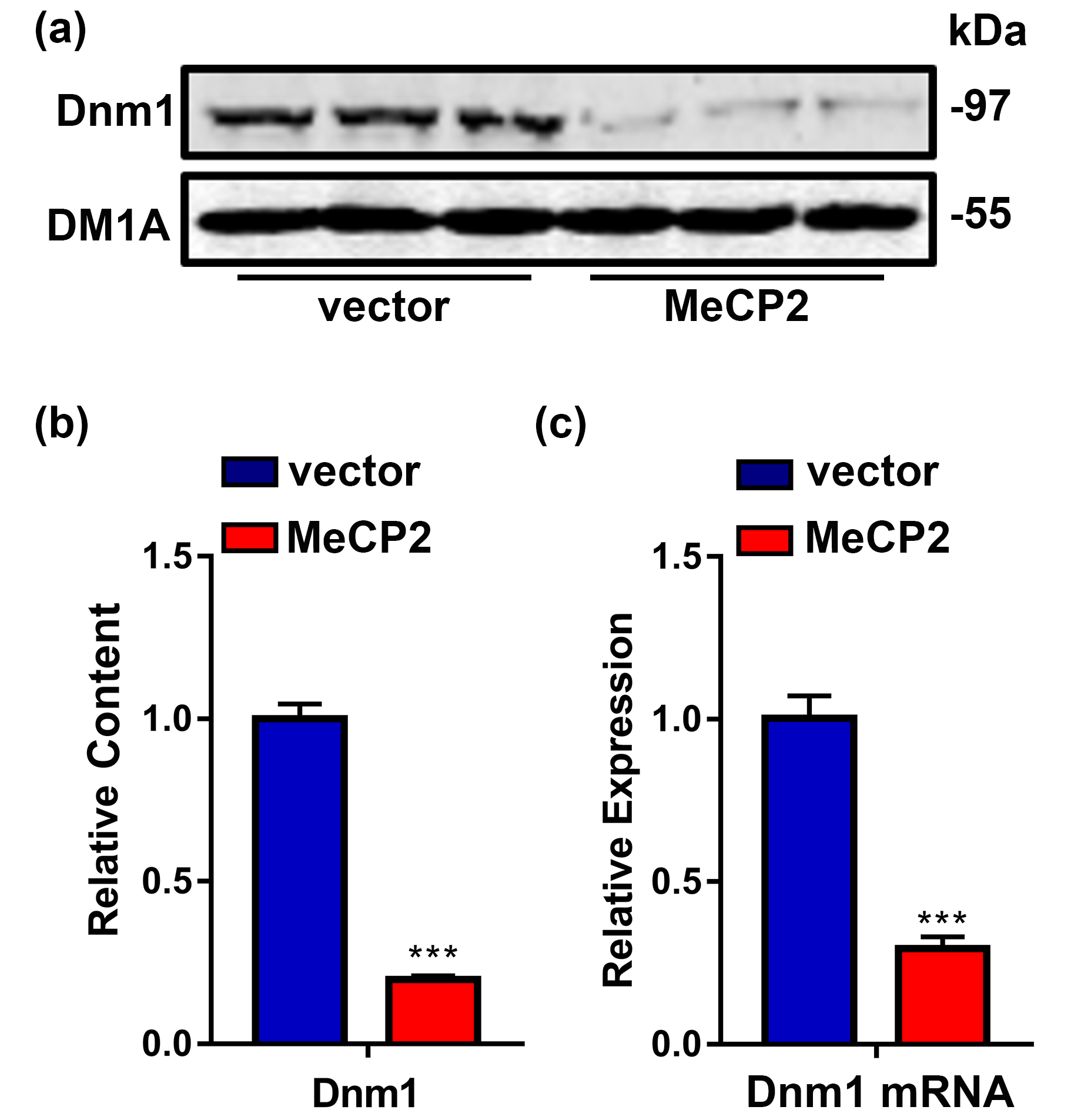


**Supplementary Figure 3. MeCP2 overexpression reduced both the mRNA and protein level of dynamin 1 in cultured neurons.**

(a) The representative blots of Dynamin1 in primary cortical neurons transfected with vector or pAAV-syn-MeCP2-GFP plasmid. (b) The quantitative analysis. (c) The mRNA level of Dynamin1 in in primary cortical neurons transfected with vector or pAAV-syn-MeCP2-GFP plasmid. N=5. ****p<0.001, vs vector.*


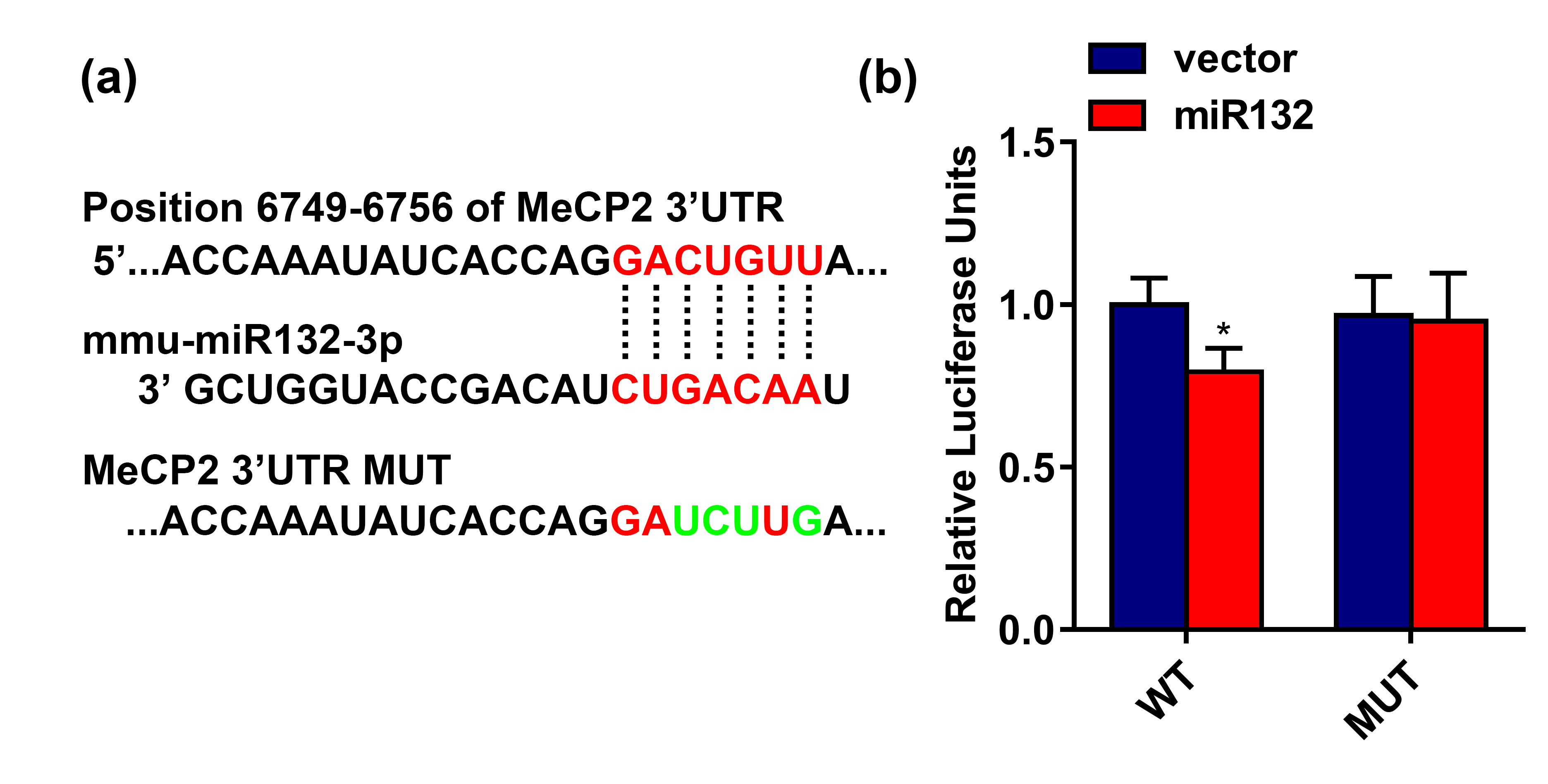


**Supplementary Figure 4. MiR-132 directly bind with the 3’UTR of MeCP2.**

(a) Predicted binding sites of miR-132 in MeCP2 3’-UTR and its mutant. (b) Human embryonic kidney 293 cells were cotransfected with miR-132 or vector with WT or mutant (MUT) 3’-UTR of MeCP2. Relative luciferase activity was assayed. N=4. **p<0.05, vs vector.*


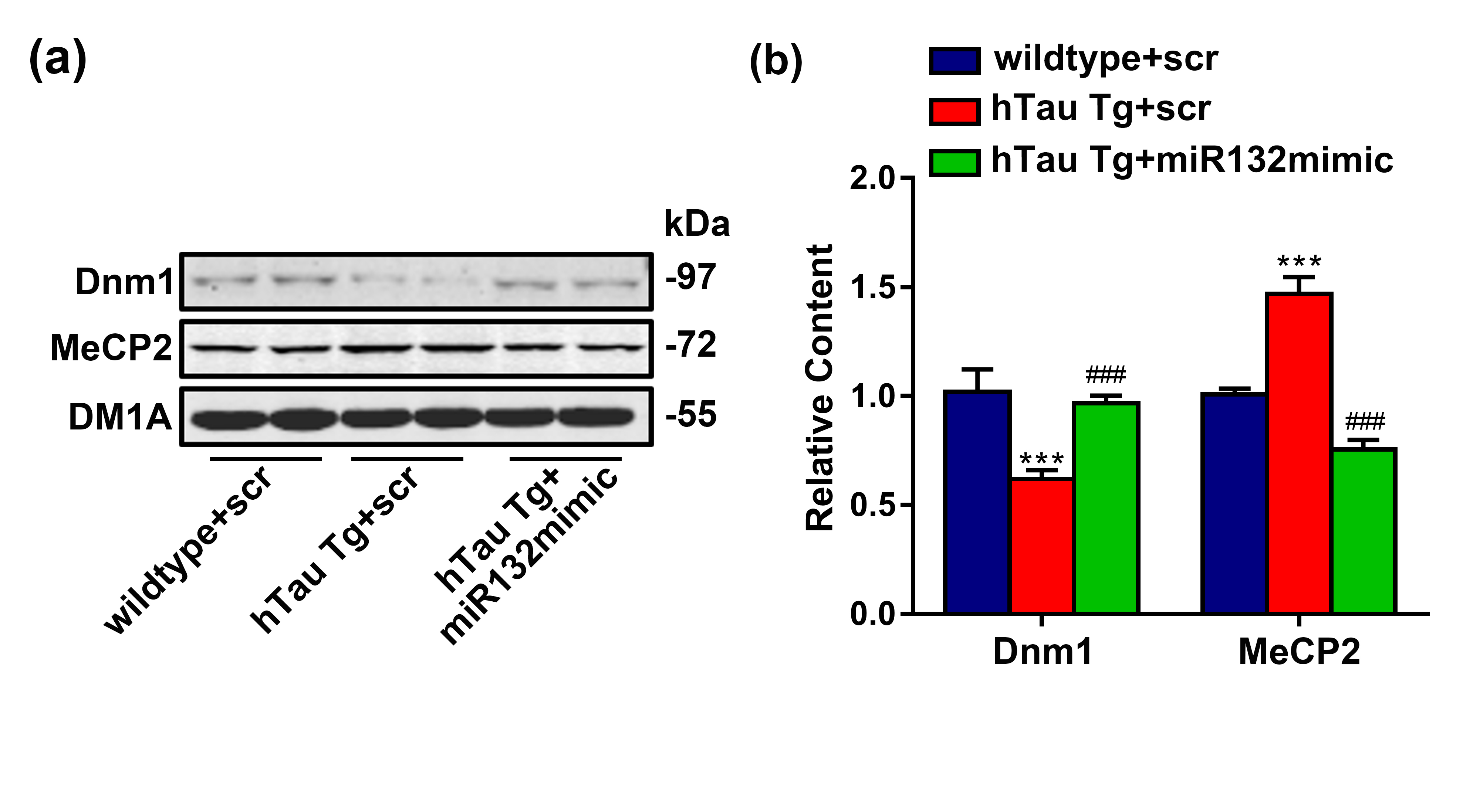


**Supplementary Figure 5. Administration of miR-132 mimics rescued the dynamin 1 loss and MeCP2 upregulation in hTau.**

(a) The representative blots of Dynamin1 and MeCP2 in the cortex of 12 weeks wildtype mice injected with mimic scramble, hTau transgenic mice injected with mimic scramble or hTau transgenic mice injected with miR132 mimics. (b) The quantitative analysis. N=6. ****p<0.001, vs wildtype+scr. ### p<0.001, vs hTau Tg mice+scr.*

**Table 1: Antibodies**

Name WB IF IHC Source/ Cat#

Dynamin 1 1:1000 Abcam ab13251

Dynamin 1 1:500 Thermo Fisher PA1-660

Dynamin 3 1:1000 Abcam ab3458

MeCP2 1:1000 Cell Signaling cst3456

Clathrin 1:1000 Proteintech 26523-1-AP

VGluT1 1:1000 Abcam ab227805

Synaptophysin 1:1000 Proteintech 17785-1-AP

pT231 1:500 Signalway

pT262 1:500 Signalway

pS396 1:1000 1:200 Signalway

pS404 1:1000 1:200 Signalway

AT8 1:1000 Signalway

Tau1 1:1000 Millipore

β-actin 1:1000 Proteintech 20536-1-AP

DM1A 1:2000 Sigma T6199

**Table2:** Human samples

Number Age (years) Gender Final Diagnosis

1. 88 M AD
2. 95 M AD
3. 81 M AD
4. 90 M [Liver](javascript:;) [cancer](javascript:;)
5. 84 M Respiratory failure
6. 87 M Multiple organ failure

**Experimental procedures**

*Animal, Antibodies and Reagents*

Human tau transgenic mice (hTau+/+) at 12 weeks and 12 months (stock number 004808) were purchased from The Jackson Laboratory. The age-matched C57BL/6J mice were supplied by the Experimental Animal Center of Tongji Medical College. All the animals were housed in a room on a 12-h light-dark cycle at 25 ± 2°C with free access to food and water. All animal experiments were carried out according to the “Policies on the Use of Animals and Humans in Neuroscience Research” revised by the Society for Neuroscience in 1995 and approved by the animal ethics committee of Huazhong University of Science and Technology. The mature sequence of scramble was UUUGUACUACACAAAAGUACUG. The antibodies are listed in Supplementary Table 1. pAAV-syn-MeCP2-GFP plasmid was friendly gifted by Qiu Zi-Long, the researcher of institute of neurology, Chinese academy of sciences.

Luciferase activity reporter assay kit was purchased from Promega. Alexa-Fluor546 Transferrin was purchased from Thermo Fisher (Tf-546, T23364); Alexa Fluor 633-conjugated secondary antibody was purchased from Thermo Fisher (A-21094).

*General clinical characteristics of study subjects*

Autopsy specimens of brain were obtained from the Tissue Bank of the Institute of Geriatrics, Chinese PLA General Hospital & Chinese PLA Medical Academy. The present study was approved by the ethics committee of Tongji Medical College (Wuhan, China). The patients’ details were showed in Supplementary Table 2.

*Surface biotinylation*

Cortex neurons were washed two times with cold PBS(pH 8.0), and incubated with 0.5 mg/ml EZ-Link Sulfo-NHS-LC-LC-Biotin (Pierce) for 25 min at 4°C for biotinylation, then washed again with PBS(pH 7.4) and incubated with 100 mM glycine in PBS for 15 min to quench the reaction. Neurons were collected in ice-cold lysis buffer, containing 50 mM Tris-HCl, pH 7.6, 150 mM NaCl, 1% NP-40, 2 mM EDTA, 1 mM sodium orthovanadate and proteinase inhibitor cocktail (Sigma,1:1000), and extracted for 30 min at 4°C. Insoluble ingredient was removed by centrifuging at 19,000 g for 20 min. The supernatants were used for immunoprecipitation. After protein concentrations were measured by BCA protein assay, the supernatants (100 μg protein) then were incubated with VGluT1 or synaptophysin antibody overnight at 4°C, followed by the addition of 20 μl of Protein A+G agarose (CW0349S, CWbiotech, China) for 3 h at 4 °C. Beads were washed four times with lysis buffer, and then Immunoprecipitates were subjected to 10% (v/v) SDS/PAGE gel for Western blotting using VGLUT1 or synaptophysin antibody.

*Luciferase Activity Assay*

Human embryonic kidney 293 cells were cotransfected with the miR-132 or the vector and wild-type or mutant MeCP2 3’-UTR plasmid. The Firefly and Renilla luciferase activities of cell lysates were detected using dualluciferase reporter assay kit (Promega). The normalized values (Renilla/firefly activity) were calculated.

*Primer sets for Q-PCR*

The primers for miR-101a, miR-181c, miR-124, miR-132 detection, and miR-132 mimics and antagomirs were purchased from GeneCopoeia. The primers used for Dynamin1: 5’-ATTTCGTGGGCAGGGAC-3′ (forward) and 5′-TGGCGGTAGGATGGGT-3’(reverse). MeCP2:5′-CCCTGGGAAACTTGTTGT-3’(forward) and 5’-CCTTGACCTCGATGCTGA-3′(reverse). Rab5: 5′-GCTAATCGAGGAGCAACAAGAC-3′(forward) and 5’-CCAGGCTTGATTTGCCAACAG-3’(reverse). Clathrin: 5’-CTTTGGCACAGGGATAGGAAAT-3’(forward) and 5’-GCTGATCTTTTTGCTTTCGGTT-3’(reverse).

*Transferrin uptake test*

The transferrin uptake test was carried out according to a previous study with minor modification . For immunofluorescence, the neurons were washed three times at 37°C with PBS and were then exposed to Tf-546 for 15 min in culture medium without B27 and Glutamax. Then, the neurons were washed with buffer containing 150 mM NaCl, 2 mM CaCl_2_, and 25 mM CH_3_COONa, pH=4.5, to remove the uninternalized Tf-546 and washed with ice-cold PBS followed by fixation using 4% paraformaldehyde. Single-round kinetics of Tf uptake and recycling were analyzed by first incubating the neurons with Tf-546 for 30 min at 0°C in medium containing Tf-546 and then washing them three times in cold PBS and incubating them in medium without Tf-546 for 0, 5, 10, 20, 40 and 50 min at 37°C. At low temperatures, Tf-546 bound to its receptor, but internalization was inhibited because endocytosis occurs only when cells are above the phase-transition temperature of the plasma membrane. Thus, the internalization of Tf-546 bound to the membrane during cold incubation was induced by transferring cells at 37°C. Dishes were then placed on ice and washed three times with cold PBS. Cells were detached from the dishes with RIPA lysis buffer and were then sonicated and centrifuged for 10 min at 10000 rpm. The fluorescence from the cell suspensions was measured.

*Stereotaxic Injection*

Mice (male wild type or hTau mice at around 12 weeks) were anesthetized using chloral hydrate (400 mg/kg, intraperitoneal). Holes were drilled above the CA3 field of the hippocampus (anterior/posterior = ±1.9 mm, medial/lateral = ±1.9 mm, dorsal/ventral = ±2.4 mm). MiR-132 mimic (100 mM, 1.5 µL) was bilaterally microinfused into the hippocampus via a cannula connected to a Hamilton (Reno, NV) microsyringe. The infusion rate was 0.2 mL/min, and the cannula was left in place for 10 minutes following completion of the infusion. After 5 days, the mice were sacrificed for following biochemical studies.

*Cell Culture*

Briefly, the cortex of the embryonic mice were collected and incubated with 0.25% trypsin in D-Hanks for 15 min, added the neuronal plating medium containing DMEM/F12 with 10% FBS to cortical suspension, and then centrifuged at 1000 × g for 5 mins. The cells were triturated and plated onto plastic culture dish, and incubated at 37°C with 5% CO_2_. Human embryonic kidney 293 cells were maintained in DMEM medium supplemented with 5% FBS, and incubated at 37°C with 5% CO2. Transfection was performed using Lipofectamine 3000 (Invitrogen, Carlsbad, CA, USA) according to the manufacturer’s instruction.

*RNA Isolation*

Total RNA was extracted by Trizol reagent (Invitrogen), and 1 μg RNA was reversely transcripted. qRT-PCR was performed on ABI Stepone Plus using SYBR Green ® Premix Ex Taq (Takara, Tokyo, Japan). MiRNA was extracted using miRNA isolation kit (Tiangen, Beijing, China).

*Western Blotting*

The cell extracts prepared from mice or cultured neurons were quantified using BCA Protein Assay Reagent (Pierce), then the protein concentration of tissues and neurons were adjusted to 4 ug/ul and 6 ug/ul with RIPA buffer (containing 1:100 PMSF and 1:1000 cocktail), respectively. Equal amounts of proteins were separated by SDS-PAGE (10%) and the proteins were transferred onto nitrocellulose membrane (Amersham, Piscataway, NJ, USA) for 1 h using the transfer apparatus (Bio-Rad, Berkeley, CA, USA). After block with 3% milk for 30 min at room temperature, the membranes were then incubated at 4 °C with primary antibodies overnight. The blots were probed by incubation with anti-rabbit or anti-mouse IgG conjugated secondary antibodies (LI-COR, Lincoln, NE, USA) for 1 hour at room temperature, and detected using the Odyssey Imaging System (LI-COR, Lincoln, NE, USA).

*Immunofluorescence*

For immunofluorescence staining, neurons were cultured on coverslips in 12-well plate. For in vivo studies, brains were fixed by transcardial perfusion of 4% paraformaldehyde and sectioned coronally. Neurons and brain sections were incubated in primary antibodies for 12-36 h at 4 °C and subsequently with AlexaFluro488- and AlexaFluro546-conjugated secondary antibodies. The image was observed with the LSM780 confocal microscope (Carl Zeiss, Oberkochen, Germany).

*Immunohistochemistry*

Mice were anesthetized with 6% chloral hydrate (0.6 mL/100 g, i.p.). 35-50 mL of normal saline was perfused through the apex cordis immediately, and 50 mL of 4% paraformaldehyde/PBS solution was subsequently used. Brain tissue was quickly dissected and postfixed in 4% paraformaldehyde/PBS solution for 24 h. Thirty-micron slices of brain tissues were cut ​​from the coronal plane with a frozen slicer (Leica, Wetzlar, Germany). The slices were incubated with primary antibody (diluted 1:200) for 36 h at 4°C, probed with biotin-labeled secondary antibodies for 1 hour at 37°C, and detected with DAB system. The images were observed under a fully automatic sectioning microscope (Olympus SV120, Tokyo, Japan).

*Virus generation*

The plasmid pEGFP-tau-2N4R, encoding hTau, was granted by Fei Liu (Jiangsu Key Laboratory of Neuroregeneration, Nantong University, Nantong, China). Based on it, lentivirus for pAOV-CAG-EGFP-MAPT was packaged by OBIO Technology (Shanghai, China).

*Statistical analysis*

Data are expressed as mean ± S.D. and analyzed using SPSS 16.0 statistical software (SPSS Inc., Chicago, IL, USA). The one-way ANOVA procedure followed by Student’s *t* tests was used to determine the differences between the groups. The significance was assessed at *p*<0.05.

**References**

Lee, Y. S., Ehninger, D., Zhou, M., Oh, J. Y., Kang, M., Kwak, C., . . . Silva, A. J. (2014). Mechanism and treatment for learning and memory deficits in mouse models of Noonan syndrome. Nat Neurosci, 17(12), 1736-1743. doi:10.1038/nn.3863

Marin, M. P., Esteban-Pretel, G., Ponsoda, X., Romero, A. M., Ballestin, R., Lopez, C., . . . Renau-Piqueras, J. (2010). Endocytosis in Cultured Neurons Is Altered by Chronic Alcohol Exposure. Toxicological Sciences, 115(1), 202-213. doi:10.1093/toxsci/kfq040

Raimondi, A., Ferguson, S. M., Lou, X., Armbruster, M., Paradise, S., Giovedi, S., . . . De Camilli, P. (2011). Overlapping role of dynamin isoforms in synaptic vesicle endocytosis. Neuron, 70(6), 1100-1114. doi:10.1016/j.neuron.2011.04.031
